# A possible role of Painless channel in the regulation of immune system activity in *Tenebrio molitor* L.

**DOI:** 10.1038/s41598-026-45339-x

**Published:** 2026-04-02

**Authors:** Natalia Bylewska, Radosław Gmyrek, Natalia Konopińska, Sara Tchórzewska, Grzegorz Nowicki, Arkadiusz Urbański

**Affiliations:** 1 Department of Animal Physiology and Developmental Biology, Faculty of Biology, Adam Mickiewicz University, Poznan, Poland; 2 genXone S.A., Złotniki, Poland

**Keywords:** Insect TRP channels, Insect cellular response, Insect humoral response, Pest control, Insect mass-rearing, Immunology, Microbiology

## Abstract

The immunomodulatory properties of transient receptor potential (TRP) channels in insects are poorly understood. Here, we provide evidence of the immunoregulatory role of one of the TRPA (transient receptor potential ankyrin) channels, Painless, in the mealworm beetle (*Tenebrio molitor*), an economically important insect species. Our research revealed that the expression patterns of *Painless* gene vary across different phases of the immune response. Additionally, we show that the cytokine Spätzle-like protein significantly influences the expression of *Painless* gene. Furthermore, we report that an allyl isothiocyanate (AITC), TRPA channel agonist which may activate the Painless channel, increases calcium level in *T. molitor* haemocytes which leading to changes in the expression of immune-related genes. On the basis of the results of the dsRNA experiment, our research also suggested that the link between Painless channel activity and immune pathways is likely mediated by Calcineurin, a calcium-activated serine/threonine phosphatase. Importantly, coinjection of AITC with *Escherichia coli* significantly improved beetle survival, indicating a functional role in host defense. The results of physiological experiments and phylogenetic analysis suggest that the modulation of Painless channel activity can be a valuable tool for pest control and large-scale *T. molitor* production.

**Supplementary Information:**

The online version contains supplementary material available at 10.1038/s41598-026-45339-x.

## Introduction

Transient receptor potential (TRP) channels are a conserved family of cation channels that participate in cell signalling. In all the phyla, TRP channels are involved in similar processes, mostly related to sensory systems and nociception^1,2^. Recently, because of their multifunctional role, TRP channels have been considered good targets for new biosafe insecticides^3^. Moreover, modulators of TRPV (transient receptor potential vanilloid) channels, one of the TRP channel families, such as pymetrozine, pyrifluquinazon, and afidopyropen, were introduced to the market.

Initially, the functions and distribution of TRP channels were connected to the nervous system, but recent findings clearly indicate their role in modulating the activity of nonneuronal cells, including immune cells^4,5^. Preliminary studies conducted by Su et al.^6^ in *Spodoptera frugiperda* suggest that TRP channels can also participate in the modulation of immune cell activity in insects, but these findings still need further confirmation.

One of the best-known insect TRP channels is Painless. Painless belongs to the TRPA (transient receptor potential ankyrin) channel family and participates in the sensory perception of different stimuli, such as nociception, thermosensation and chemosensation, and its expression has been observed in different sets of cells^7,8^. The activation of Painless channel by environmental factors such as noxious heat or its agonists increases the level of intracellular calcium, one of the most important secondary messengers and modulators of cell activity^9^. Research by Su et al.^6^ revealed that *Painless* gene is likely also expressed in haemocytes and that the expression level of *Painless* gene was upregulated in *S. frugiperda* haemocytes 24 h after bacterial infection. However, these preliminary data did not provide strong evidence confirming the direct immunomodulatory role of Painless channel. For this reason, the main aim of our research was to analyse the possible influence of Painless channel on insect immune system, on example of mealworm beetle *Tenebrio molitor*.

The choice of model organism was not accidental. *Tenebrio molitor* is a beetle from the Tenebrionidae family that is considered a serious pest whose activity has led to the destruction of 15% of the global production of stored grains^10,11^. Owing to its easy maintainability under standardized conditions and well-characterized physiology, including immune system function, along with the availability of genomic and transcriptomic data, *T. molitor* is being increasingly considered a model insect organism in ecotoxicological and immunological research^12–15^. Paradoxically, although *T. molitor* is considered a pest species, its ease of breeding and favourable body composition make it a potential source of proteins and fats for multiple sectors, ranging from food and feed production to the cosmetic and petrochemical industries^16^. For this reason, a better understanding of the biology and physiology of mealworm beetles, including the regulation of their immune system activity, is needed.

During our research, we raised several important questions that we attempted to answer. The first question concerns whether the expression level of the *Painless* gene changes during *T. molitor* immune system activation by different immune stimulators. To address this, we analysed the expression pattern of the *Painless* gene in various parts of the nervous system and in immune‑related tissues and cells (haemocytes and fat body) of *T. molitor*. The next step involved investigating the potential role of Painless channel activation by an agonist in modulating calcium levels in haemocytes, which are among the most important immune cells. An increase in the intracellular calcium level is directly associated with the activity of TRP channels and is considered a secondary messenger crucial for regulating cellular activity. However, our aim was not only to determine whether activation of the Painless channel influences immune cell activity but also to determine how it does so. Therefore, a separate objective was to analyse the immune‑modulatory effects associated with Painless channel function. For this purpose, we examined immune parameters such as the number of circulating haemocytes and changes in the expression levels of immune‑related genes. This part of the study was further expanded by assessing immune mechanisms after both agonist‑induced activation and efficient knockdown of the *Painless* gene.

**Fig. 1:**
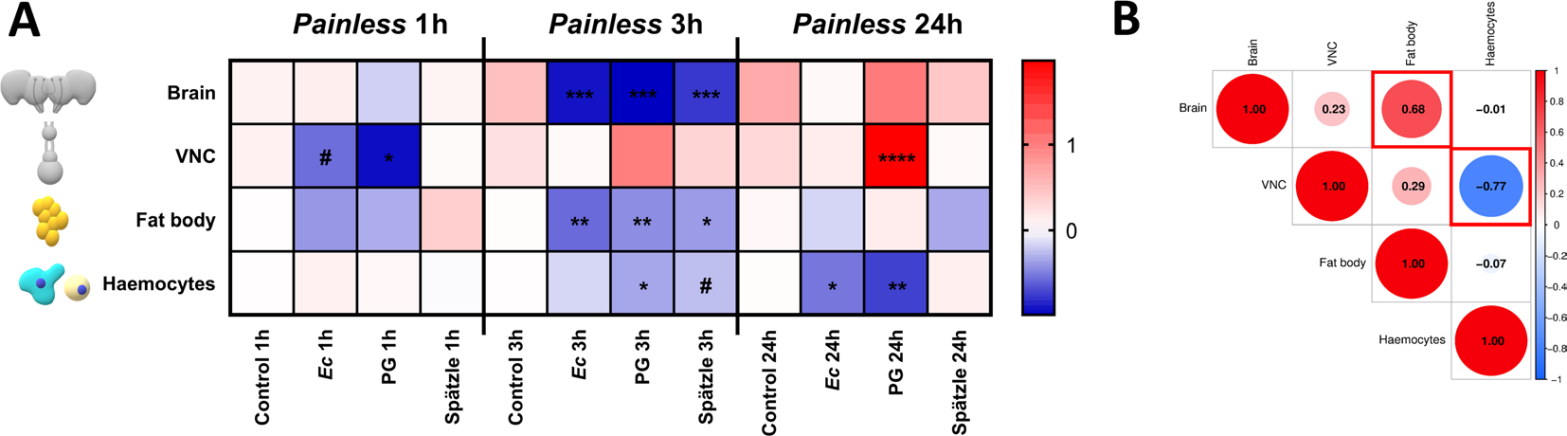
The expression patterns of *Painless* gene (**A**) in the *Tenebrio molitor* brain, ventral nerve cord (VNC), fat body and haemocytes after activation of the immune system via injection of 2 µL of *Escherichia coli* suspension (*Ec*, 1 mg/1 mL in physiological saline, 1.27 × 10^6^ cell/µL), *Staphylococcus aureus* peptidoglycan suspension (PG, 1 mg/1 mL in physiological saline), and Spätzle-like peptide at concentration of 10^− 7^ M, one of the most important insect cytokines. Control – individuals injected with physiological saline (PS). Samples were collected at three different time points, 1, 3 and 24 h after the injection. For better visualization of the results, the presented values are expressed as log2fold values. Different colours indicate different gene expression levels. Shades of blue, downregulation; shade of red, upregulation. Asterisks indicate significant differences compared with control individuals; #0.05 ≤ *p* ≤ 0.1; *p ≤ 0.05, **p ≤ 0.01, ***p ≤ 0.001, ****p ≤ 0.0001. (**B**) Correlation matrix showing the relationships among the expression levels of *Painless* gene in the brain, VNC, fat body and haemocytes after activation of the immune system. The dot size and the number inside indicate the r value. Red brackets indicate statistically significant correlations (*p* ≤ 0.05). Different colours indicate different *r* values. Shades of blue indicate positive correlations (*r* ≥ 0), and shades of red indicate negative correlations (*r* ≤ 0). To estimate the correlation of the data, the Pearson correlation coefficient method was used. The matrix was generated via SRplot software (https://www.bioinformatics.com.cn/srplot).

By evaluating the immunomodulatory role of Painless channels in insects, we enhance our knowledge of the basis of the regulation of the innate immune response and its evolution. Moreover, as long as TRP channel modulators are considered insecticides, research concerning the Painless influence on pest species physiology provides more insight into their mode of action. Additionally, owing to the U-turn in *T. molitor* perception as a source of proteins and fats, our research can reveal how to modulate the *T. molitor* immune system to improve their survival and obtain feed characterized by better quality, which might be linked to higher antimicrobial peptide (AMP) concentrations (Fig. 1).

## Results

### Expression pattern of *Painless* gene after immune system activation

The first step was the determination of the expression patterns of *Painless* gene in the nervous system (brain and ventral nerve cord (VNC)) and cells closely associated with the immune response (fat body and haemocytes) after the activation of the *T. molitor* immune system, including the cytokine Spätzle-like peptide (*Tm*Spz-like).

**Fig. 2:**
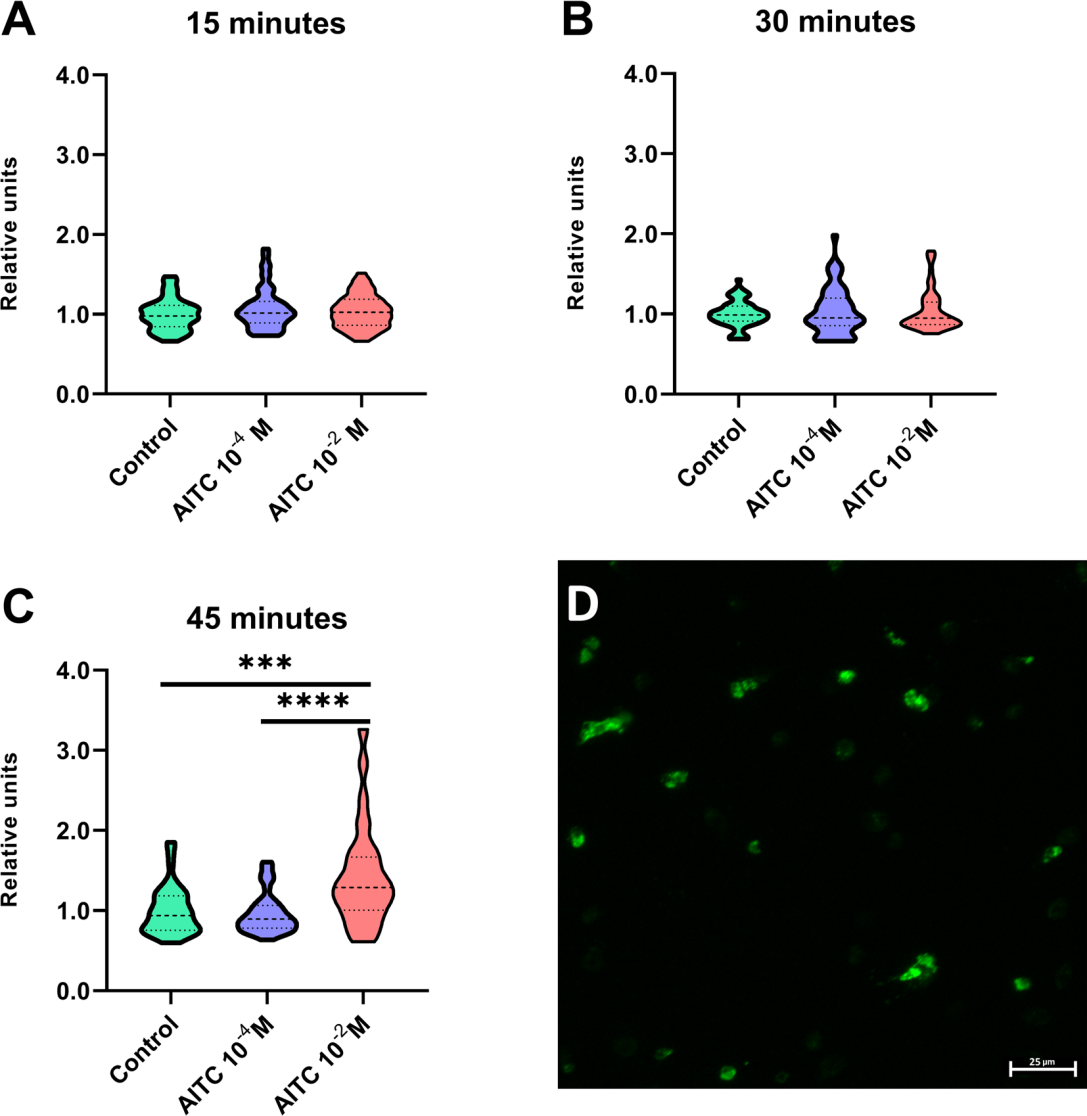
Analysis of the relative concentration of Ca^2+^ in haemocytes collected from *Tenebrio molitor* and treated with allyl isothiocyanate (AITC) *in vitro*. The micrographs were taken after 15, 30–45 min of incubation with AITC at concentrations of 10^− 4^ and 10^− 2^ M (solvent: 0.5% ethanol in physiological saline). Control – haemocytes incubated with 0.5% ethanol in physiological saline. Violin plots present the distribution of data points. D- Representative micrograph of haemocytes stained with Fluo-8 AM; green – calcium inside haemocytes; *** *p* ≤ 0.001, ****p ≤ 0.0001.

The *Painless* gene was differentially expressed in nervous system- and immune-related cells (Fig. 2 and Fig. S1). One hour after the injection of immune system activators, significant changes were observed only in the VNC after *Staphylococcus aureus* peptidoglycan (PG); however, strong downregulation of *Painless* was also observed after *Escherichia coli* suspension (*Ec*) treatment.

In the three-hour variant, the changes in the expression level of *Painless* gene were more pronounced. Interestingly, in the nervous system, the expression of the *Painless* gene was strongly downregulated after the injection of all immune system activators. However, these changes were visible only in the *T. molitor* brain. Similar changes and significant downregulation of *Painless* were reported in the fat body three hours after the injection of *Ec*, PG or *Tm*Spz-like. On the other hand, a significant decrease in the expression level of the gene encoding Painless channel was observed in haemocytes only three hours after PG injection. However, a strong response was also reported after *Tm*Spz-like treatment.

Twenty-four hours after the activation of *T. molitor*, the immune response clearly changed in the direction of the observed results. But, statistically significant changes were noted only in the VNC 24 h after the injection of PG. In the case of immune-related cells, no significant modulation of *Painless* expression was detected in the fat body. Nevertheless, in haemocytes *Painless* gene was significantly downregulated after *Ec* and PG treatment.

An additional analysis of the Pearson correlation coefficient revealed interesting relationships between the expression of *Painless* gene in different cells after the activation of the *T. molitor* immune system. There was a significant positive correlation between the expression level of *Painless* in the brain and that in the fat body (*r* = 0.68, *p* ≤ 0.05). On the other hand, the expression levels of *Painless* gene in VNC and haemocytes were negatively correlated (*r* = − 0.77, *p* ≤ 0.05).

### Effect of Painless channel activation on calcium level in haemocytes

Research by Su et al.^6^ and our findings highlight the strong connection between the action of TRP channels and insect haemocytes. For this reason, our next step was to evaluate the possibility of haemocyte activation via the potential stimulation of Painless channels. One of the indicators of cell activation is an increase in the calcium level in the cell via calcium influx or its release from the endoplasmic reticulum^17^. For this reason, to verify the hypothesis concerning the participation of Painless channels in the regulation of haemocyte activity, the direct effects of allyl isothiocyanate (AITC), a potent agonist of TRPA channels, including Painless channel, on the calcium concentration in haemocytes were determined. Owing to the kinetic changes in the calcium level in haemocytes, the relative calcium concentration was analysed after 15-, 30- and 45-minute incubations of haemocytes in solutions of physiological saline (PS) and AITC at concentrations of 10^− 4^ and 10^− 2^ M^18^. Haemocytes were chosen as primary cells for calcium experiments to obtain a clean monolayer of immune-related cells. Additionally, haemocytes are considered central immune cells in insects; which are important for shaping cellular and innate immunity and are functional and morphological homologues of vertebrate immune cells^19^.

Analysis of micrographs of haemocytes after incubation with AITC revealed that the relative calcium concentration inside the haemocytes was significantly greater after 45 min of treatment (Fig. 2). At this time point, the calcium concentration significantly increased after 10^− 2^ M AITC treatment compared with that in control individuals (Dunn’s multiple comparisons test, *p* ≤ 0.001) and in haemocytes treated with 10^− 4^ M AITC (Dunn’s multiple comparisons test, *p* ≤ 0.0001).

### Influence of the modulation of Painless activity on basic immune parameters

#### Circulating haemocyte count (CHC)

**Fig. 3:**
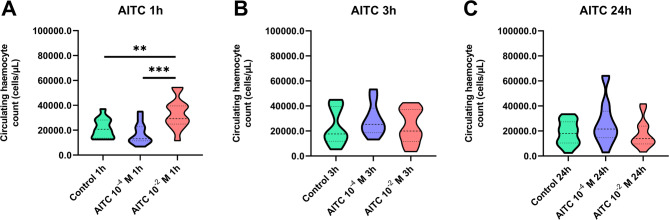
Circulating haemocyte count (CHC) at 1, 3–24 h after the injection of allyl isothiocyanate (AITC) at concentrations of 10^− 4^ and 10^− 2^ M (solvent: 0.5% ethanol in physiological saline). Control – beetles injected with 0.5% ethanol in physiological saline. The violin plot presents the data distribution.  **p ≤ 0.01, ***p ≤ 0.001.

With respect to *T. molitor* haemocytes, the next step in our research was the analysis of the effects of Painless channel activation via AITC on the circulating haemocyte count (CHC), one of the basic immune parameters. On the basis of the dynamics of the haemocyte response, the CHC value was estimated at three time points: 1, 3 and 24 h after the injection of 10^− 4^ or 10^− 2^ M AITC.

The CHC analysis revealed the influence of potential Painless channels activation on the number of circulating haemocytes. However, significant changes were observed only one hour after the injection of AITC. At this time point, AITC application caused a significant increase in the CHC value in comparison with that of the control beetle, but only at a concentration of 10^− 2^ M (Dunnett’s multiple comparisons test, *p* ≤ 0.01). Additionally, significant differences were observed between 10^− 4^ M AITC and 10^− 2^ M AITC (Dunnett’s multiple comparisons test, *p* ≤ 0.001) (Fig. 3).

#### Changes in the expression levels of immune-related genes

**Fig. 4:**
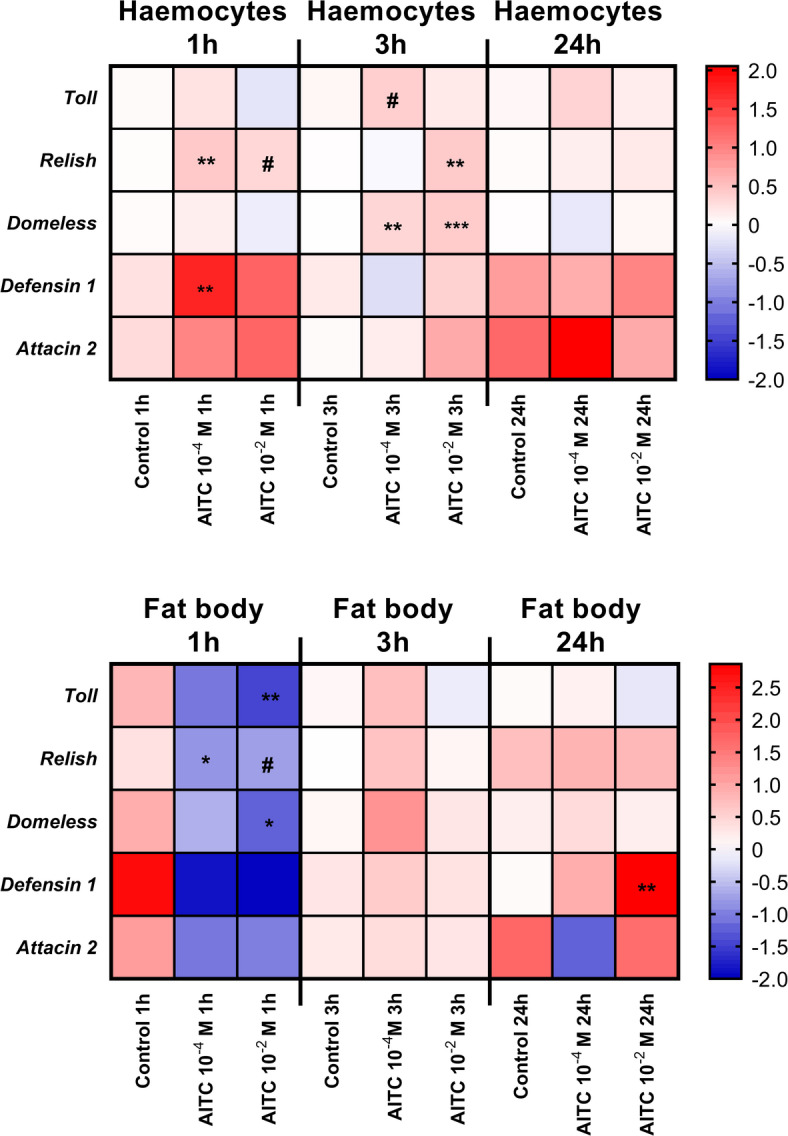
Expression patterns of immune-related genes in the haemocytes and fat body of *Tenebrio molitor* after injection of a solution of allyl isothiocyanate (AITC) at concentrations of 10^− 4^ and 10^− 2^ M (solvent: 0.5% ethanol in physiological saline). Control – individuals after injection of 0.5% ethanol in physiological saline. Samples were collected 1, 3 and 24 h after the injection of AITC. For better visualization of the results, the presented values are expressed as log2fold values. Different colours indicate different gene expression levels. Shades of blue, downregulation; shade of red, upregulation. Asterisks indicate significant differences compared with control individuals; # 0.05 ≤ *p* ≤ 0.1; * *p* ≤ 0.05, ** *p* ≤ 0.01, *** *p* ≤ 0.001.

To better understand the changes observed in immune cells (haemocytes and fat body) associated with the potential activation of Painless channels, the changes in the expression levels of immune-related genes in haemocytes and fat body after AITC injection were analysed. For this purpose, we selected genes associated with the main immune pathways (*Toll* – Toll pathway, *Relish* – Imd pathway, and *Domeless* – JAK/STAT pathway) and genes encoding two antimicrobial peptides (*Defensin 1* and *Attacin 2*).

Molecular analysis of haemocytes and fat body revealed a strong tissue-specific response to AITC (Fig. 4, Fig. S2 and S3). Compared with those in control individuals, the expression levels of the tested immune genes in haemocytes generally tended to increase. Interestingly, one hour after the injection of 10^− 4^ M AITC, significant overexpression of the *Relish* and *Defensin 1* genes was observed. However, the changes in the expression levels of these genes in haemocytes were more apparent three hours after AITC injection. At this time point, significant overexpression of *Relish* (10^− 2^ M AITC) and *Domeless* (10^− 2^ M and 10^− 4^ M AITC) was reported. Interestingly, the *Toll* gene was noticeably overexpressed three hours after 10^− 4^ M AITC injection. In the 24-hour variant, no significant changes in the expression of immune-related genes were observed.

In samples of *T. molitor* fat body, the general observed trend was opposite to that reported for haemocytes (Fig. 4 and Fig. S3). One hour after AITC treatment, the downregulation of immune-related genes was apparent. Interestingly, the expression of *Toll* (10^− 2^ M AITC), *Relish* (10^− 4^ M AITC) and *Domeless* (10^–2^ M AITC) significantly differed from that of the control individuals. In terms of the *Relish* expression level, a noticeable downregulation was also observed in the 10^− 2^ M AITC treatment group. Compared with the changes in haemocytes, significant changes in the expression of immune-related genes were not observed three hours after the application of AITC. In the 24-h variant, the only significant change was the strong overexpression of *Defensin 1* after 10^− 2^ M AITC treatment.

**Fig. 5:**
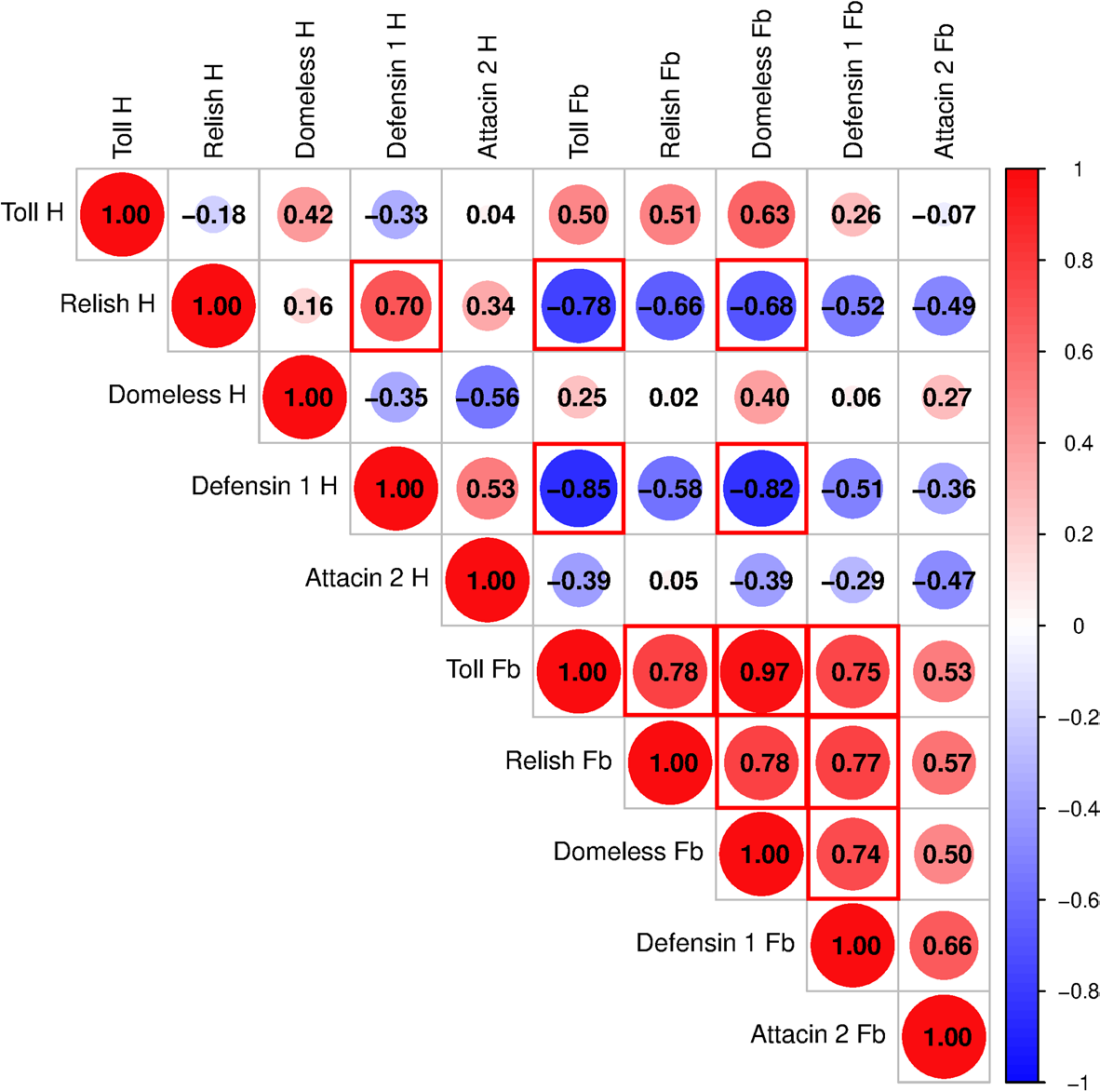
Correlation matrix showing the relationships between the expression levels of immune-related genes in haemocytes and the fat body of *Tenebrio molitor* after the injection of allyl isothiocyanate (AITC). The dot size and the number inside indicate the r value. Red brackets indicate statistically significant correlations (*p* ≤ 0.05). Different colours indicate different r values. Shades of blue indicate positive correlations (*r* ≥ 0), and shades of red indicate negative correlations (*r* ≤ 0). To estimate the correlation of the data, the Pearson correlation coefficient method was used. The matrix was generated via SRplot software (https://www.bioinformatics.com.cn/srplot).

The Pearson correlation coefficient analysis of the molecular data after AITC treatment revealed strong and significant correlations between the expression of immune-related genes, which may indicate orchestration of immune system activity after potential activation of Painless channels (Fig. 5). Additionally, in this statistical analysis, differences between the haemocyte and fat body samples were detected. The expression of *Relish* and *Defensin 1* in haemocytes was negatively correlated with the expression of *Toll* and *Domeless* in the fat body. On the other hand, the expression of haemocyte *Relish* was positively correlated with the expression of *Defensin 1* in these cells. In terms of the expression of immune-related genes in the fat body, the expression of fat body *Toll*, *Relish* and *Domeless* genes was positively intercorrelated. Additionally, the expression of these genes was positively correlated with the expression of fat body *Defensin 1* (Fig. 5).

#### Effect of *Painless* knockdown on the *T. molitor* immune system

**Fig. 6:**
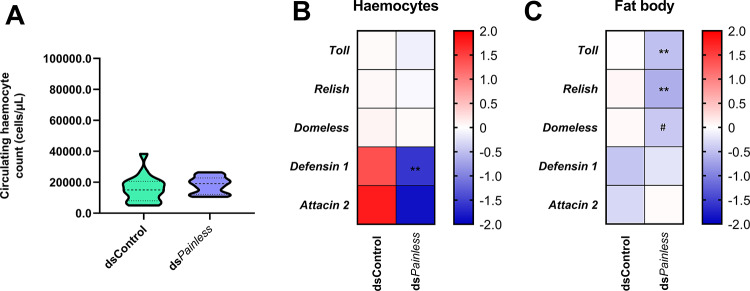
Physiological effects of *Painless* knockdown via dsRNA. (**A**) Violin plot of the circulating haemocyte count experiment (**B** and **C**) Expression levels of immune-related genes in haemocytes and fat body after knockdown of *Painless* gene via dsRNA injection (2 µg of dsRNA in DNA/RNA-free water). dsControl – beetles injected with a dsRNA-targeted gene encoding *Galleria mellonella* lysozyme (*GmLys*) (2 µg of dsRNA in DNA/RNA-free water). To enhance the clarity of the results, the data are shown as log2-fold change values. Gene expression levels are represented using different colours: blue tones indicate downregulation, whereas red tones indicate upregulation. ** *p* ≤ 0.01; # 0.05 ≤ *p* ≤ 0.1.

To confirm the effect of the potential activation of Painless channels on the *Tenebrio* immune system, the analysis of the tested immune parameters was repeated after the knockdown of *Painless* gene via dsRNA experiments.

Although ds*Painless* treatment did not affect the number of circulating haemocytes, the significant results in the expression of immune-related genes in haemocytes and the fat body were observed (Fig. 6A). In haemocyte samples after the injection of ds*Painless*, significant downregulation of the *Defensin 1* gene was observed (Mann‒Whitney U test, U = 4.00, *p* ≤ 0.05) (Fig. 6B, and Fig. S4). On the other hand, in fat body, injection of ds*Painless* caused a significant decrease in the expression levels of the *Toll* and *Relish* genes compared with those in control individuals (*Toll* - Mann‒Whitney U test, U = 4.00, *p* ≤ 0.01; *Relish* - Student’s *t*-test, *t *= 3.35, *p *≤ 0.01). Additionally, the expression of the *Domeless* gene was strongly downregulated after dsRNA-based knockdown treatment (Mann‒Whitney U test, U = 29.50, *p* = 0.08) (Fig. 6C, and Fig. S4).

#### Calcineurin as a potential linker between calcium level and immune pathways

On the basis of previous results, we can assume that the activation of TRPA channels, including Painless channel, may increase the relative calcium level in *Tenebrio* haemocytes. Furthermore, we revealed changes in the expression levels of immune-related genes after AITC treatment and dsRNA-based knockdown of *Painless* gene. However, the relationship between enhanced calcium level and immune cell activity remains unclear. Research conducted by Wei et al.^20^ and Mencarelli et al.^21^ reported that a crucial linker between calcium level and immune pathways can be Calcineurin, a calcium-activated serine/threonine phosphatase. Therefore, the next step in our project was the determination of changes in the expression level of *Calcineurin* in immune-related cells after AITC and ds*Painless* treatment.

**Fig. 7:**
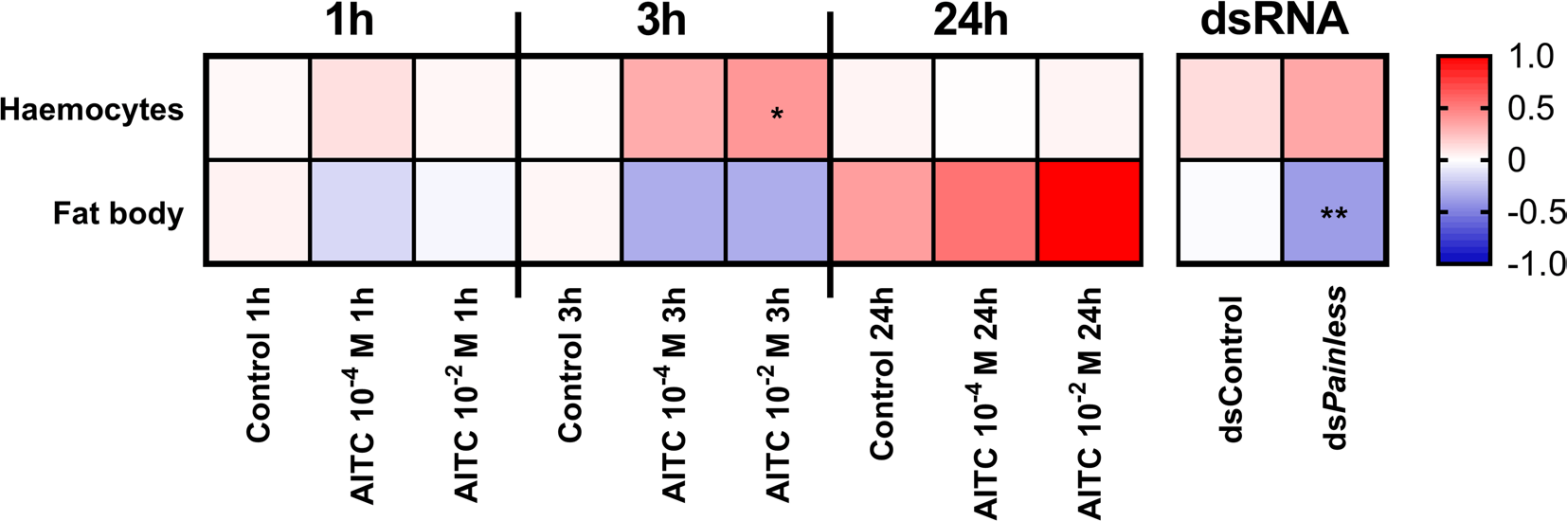
Expression patterns of the *Calcineurin* gene in haemocytes and fat body of *Tenebrio molitor* after injection of allyl isothiocyanate (AITC) at concentrations of 10^− 4^ and 10^− 2^ M and dsRNA-based knockdown of *Painless* gene. Control – individuals after injection of physiological saline. Samples were collected 1, 3 and 24 h after the injection of AITC. dsControl – beetles injected with a dsRNA-targeted gene encoding *Galleria mellonella* lysozyme (*GmLys*) (2 µg of dsRNA in DNA/RNA-free water). For better visualization of the results, the presented values are expressed as log2fold values. ds*Painless* – individuals injected with dsRNA targeting the *Painless* gene (2 µg of dsRNA in DNA/RNA-free water). Different colours indicate different gene expression levels. Shades of blue, downregulation; shade of red, upregulation. Asterisks indicate significant differences compared with control individuals; * *p* ≤ 0.05, **p ≤ 0.01.

The results of the molecular analysis revealed statistically significant overexpression of *Calcineurin* in the haemocytes collected after 10^− 2^ M AITC treatment (Dunnett’s multiple comparisons test, *p* ≤ 0.001). In addition, after knockdown of *Painless* gene, significant downregulation was reported in the fat body (Student’s *t*-test, *t* = 3.294, *p* ≤ 0.01) (Fig. 7, and Fig. S5).

### Effects of AITC injection and knockdown of *Painless* gene on *T. molitor* lifespan

**Fig. 8:**
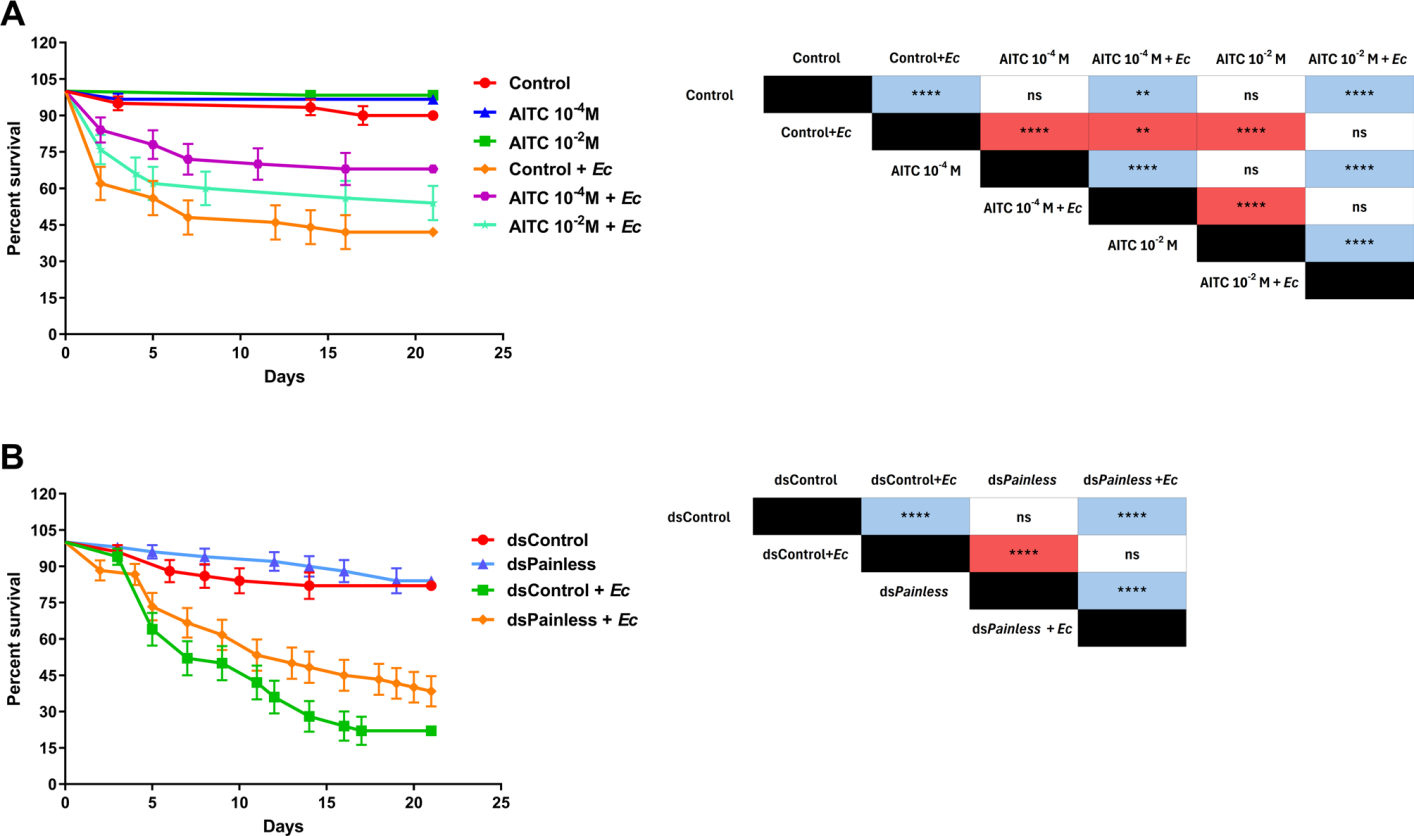
Survival analysis of *Tenebrio molitor* after injection of AITC (**A**) or dsRNA targeting *Painless* gene (**B**). The control for Graph A represents individuals injected with 0.5% ethanol in physiological saline. AITC – individuals injected with allyl isothiocyanate (AITC) at concentrations of 10^− 4^ and 10^− 2^ M (solvent: 0.5% ethanol in physiological saline). dsControl – control individuals for the dsRNA experiment; beetles were injected with the dsRNA-targeted gene encoding *Galleria mellonella* lysozyme (*GmLys*) (2 µg of dsRNA in DNA/RNA-free water). ds*Painless –* beetles after the knockdown of *Painless* genes (injection of 2 µg of dsRNA in DNA/RNA-free water). For all the research variants (A and B), additional treatment with 2 µL of a suspension of *Escherichia coli* (*Ec*) in physiological saline was performed (1 mg/mL, 1.27 × 10^6^ cells/µL). Table – Statistical comparison of estimated survival curves based on the Gehan-Breslow‒Wilcoxon test; ns – nonsignificant difference, ** *p* ≤ 0.01, **** *p* ≤ 0.0001; *n* ≤ 50.

Despite the physiological changes observed mostly at the molecular level, the most important question is whether the modulation of Painless channels affects the survival of *T. molitor* during the stress response, including pathogen infection. For this purpose, we evaluated the survival of *T. molitor* not only after a single injection of AITC or ds*Painless* but also after activation of the *T. molitor* immune system via *E. coli* injection. The pathogen was chosen on the basis of our previous research, which revealed its significant impact on *T. molitor* mortality^22^ (Fig. 8A).

Compared with the control individuals, the injection of 10^− 4^ or 10^− 2^ M AITC had a minor influence on the survival of *T. molitor*. In these groups, the survival ratio oscillated above 90%, and no statistically significant differences were observed. The effect of AITC injection was more pronounced during the activation of the *T. molitor* immune system via *E. coli* injection. The injection of AITC just before the application of *E. coli* increased the survival of *T. molitor* beetles. However, statistically significant differences compared with the control were observed only in the 10^− 4^ M AITC treatment (10^− 4^ M AITC + *Ec*). Interestingly, compared with the control, the knockdown of *Painless* gene did not affect *T. molitor* survival. Additionally, no significant differences were observed after activation of the *T. molitor* immune system by *E. coli* (Fig. 8B).

### Phylogenetic analyses

**Fig. 9:**
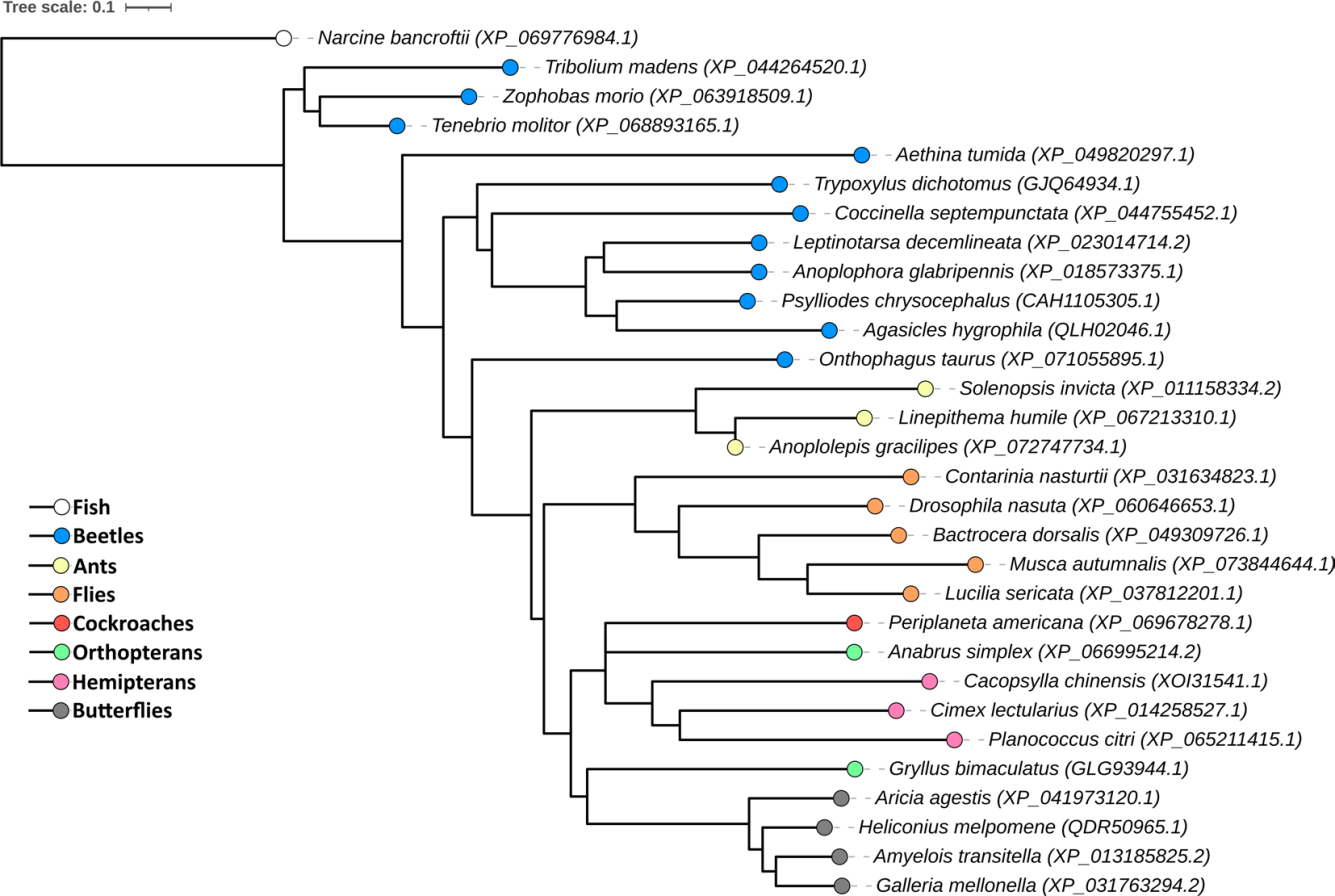
Phylogenetic tree of Painless channel sequences of selected insect and vertebrate species. The data were retrieved from an available database (https://www.ncbi.nlm.nih.gov). A phylogenetic tree was constructed with NCBI Tree Viewer (https://www.ncbi.nlm.nih.gov/tools/treeviewer/). The phylogenetic distance was calculated via the Grishin method (fast minimum evolution, max sequence difference = 0.85). The different colours indicate different insect or vertebrate groups. In brackets – NCBI sequence ID. Distance scale - the differences between sequences (0.1 = 10%). The tree was visualized with iTOL software (https://itol.embl.de).

One of the most important questions during the development of new pest strategies, which might also be important for improving the insect mass rearing process, is the specificity of new tools. Undoubtedly, differences in the sequences of targeted proteins between target species and other animals, especially pollinators, are very helpful for this process. For this purpose, we conducted a phylogenetic analysis of Painless protein sequences among representatives of different insect orders.

The phylogenetic analysis of Painless protein sequences revealed very interesting results. The proteins identified as Painless or Painless-like protein in Tenebrionidae beetles (*Tribolium madens*, *Zophobas morio* and *T. molitor*) were assigned to clusters separated from other beetle species (Fig. 9). Despite these results, in the ‘Tenebrionidae cluster’, the percentage of identity varies between 60.25% (*Z. morio*) and 50.25% (*T. madens*), but the query cover value does not fall below 98%. A comparison of the sequences of *T. molitor* Painless with those of other beetle species revealed that the percentage of identity varied from 29.04% to 25.88%.

The representatives of other insect orders were assigned together with separate clusters. These results point to the existence of structural differences in the Painless channels in different orders. Moreover, comparisons with *T. molitor* from other insect orders revealed visible differences in the sequence identity and query coverage of Painless protein. For example, for representatives of butterflies, *Galleria mellonella* (% identity − 27.17%; query cover − 56%), hemipterans *Planococcus citri* (% identity − 27.03; query cover − 41%), dipterans – *Drosophila nasuta* (% identity – 25,17%; query cover – 17%) and hymenopterans *Solenopsis invicta* (% identity 28.77%; query cover – 32%). Interestingly, a similar percentage of identity and query cover value was noted in comparison with *T. molitor* Painless to the TRPA1 channel of the fish *Narcine bancroftii* (% identity – 28.71%, query cover − 25%).

## Discussion

Here, we present new findings concerning the possible role of one of the most important TRP channels, Painless, in the regulation of *T. molitor* immune system activity, which may be useful for the development of novel pest control strategies. On the other hand, the physiological effects, especially those observed after AITC treatment, suggest the potential use of TRPA channel modulators in insect mass rearing.

The first step in our research was to confirm whether *Painless* gene expression changed after the activation of the immune system of *T. molitor*. The results revealed interesting time switches between the expression of *Painless* in the different parts of the nervous system and immune-related cells. Statistically significant changes in the expression level of *Painless* gene were observed not only after *Ec* and PG treatment but also after injection of *Tm*Spz-like. These findings suggest that pathways related to Painless channels may be modulated not only by pathogens but also by cytokines associated with the immune response. To our knowledge, these findings are the first to highlight the possible relationships between insect cytokines and TRP channels.

On the basis of the data related to the dynamic response mediated by TRP channels, strong and significant downregulation of *Painless* genes may indicate the inhibition of pathways associated with this channel and a reduction in the overresponse of the tested cells/tissue. Moreover, the time switch between the changes in the expression level of *Painless* gene may be the result of differences between local (VNC) and systemic (brain) responses. Interestingly, the downregulation of *Painless* gene was prolonged in haemocytes to 24 h. On the basis of the immunostimulatory effect of AITC on *T. molitor* haemocytes, the changes in the expression pattern of *Painless* gene may be linked to the inhibition of immune system activity in the later phase of the immune response. However, our results contrast with those of Su et al.^6^, who reported that *Painless* expression was significantly upregulated in the haemocytes of *S. frugiperda* larvae 24 h after the injection of *Serratia marcescens.* The observed differences may be associated with the different insect species, developmental stages, and immune system activators, highlighting the need for detailed investigations of the role of Painless channels in representatives of different insect orders.

After the affirmative answer to the question of whether *Painless* gene expression changed after the activation of the *T. molitor* immune system, the next step was to evaluate whether the potential activation of TRPA channels, including Painless channels, influences *T. molitor* immune cells directly via changes in the intercellular calcium level. Overall, TRPA channel activity is inextricably linked to calcium signalling. For example, research conducted on mouse T lymphocytes, which express TRPA1 channels, revealed a rapid increase in calcium level after the application of AITC at concentration of 10^− 4^ M. This state persisted throughout the entire duration of the experiment (half an hour)^23^. Interestingly, increased calcium level in T lymphocytes was not only the result of calcium influx but also the result of calcium release from the endoplasmic reticulum^23^. On the other hand, research conducted on haemocytes of the mollusc *Patinopecten yessoensisna* revealed that the intracellular level of calcium increases in these cells three hours after the application of AITC at a concentration of 2.02 × 10^− 1^ M^24^. A comparison of these examples with our results revealed that in the case of *T. molitor* haemocytes, we obtained intermediate results. A statistically significant increase in calcium level was observed only after 45 min of haemocytes incubation with 10^− 2^ M AITC. These results suggest that the observed changes in the calcium level in *T. molitor* haemocytes were secondary effects. Research on megakaryocytes has revealed that TRPA1 channels participate in the regulation of the store-operated calcium entry (SOCE) mechanism, especially with respect to stromal interaction molecule 1 (STIM1), a key protein that regulates the internal calcium level^25^. For this reason, increasing the calcium level at later time points may be an effect of the release of calcium ions from the endoplasmic reticulum. The observed changes may also be related to the cooperation of Painless with other TRP channels, such as Nanchung, Inactive or Pyrexia, but this hypothesis needs further investigation. Additionally, we cannot exclude the possibility that the observed changes at later time points may also be the result of the chosen method, and more sensitive real-time analysis allows significant changes to be observed a few minutes after AITC application. Notably, current research suggests that AITC can also activate other TRPA channels in insects, including TRPA1^26^. Therefore, to fully confirm the influence of Painless channel on calcium level in *T. molitor* haemocytes the additional research is needed, for example, on the basis of the CRISPR-Cas9 method or research assuming the development of highly specific agonists and antagonists of Painless channel.

Although the changes in calcium level points to immune cell activation, these results did not answer the question of whether the Painless channels can influence immune cell functions. For this purpose, we examined the CHC value and changes in the expression of immune-related genes after the potential activation of Painless via AITC and the knockdown of *Painless* gene.

The application of AITC caused an increase in the number of circulating haemocytes one hour after 10^− 2^ M AITC treatment. The increase in the CHC value after AITC injection may be related to the direct effect of activating TRPA channels, including Painless channel, and stimulating cell proliferation by increasing the calcium level. For example, research by Schilling et al.^27^ revealed that inhibition of TRPM (transient receptor potential melastatin) channels via antagonists abolished macrophage proliferation. However, the time point (one hour) at which significant changes were observed suggested that the increase in the CHC value is connected to the realising to haemolymph of adhered haemocytes rather than their proliferation. Notably, we did not exclude the possibility that the indirect effect of TRPA channel activation was related, for example, to hormonal regulation of the stress response. Numerous studies have shown that insect hormones participate in the response to stress factors, such as octopamine, adipokinetic hormones (AKHs) or tachykinin-related peptides (TKRPs), increasing the number of circulating haemocytes^28^. As in vertebrates, current data and hypotheses suggest that TRP channels are also associated with the release of neuropeptides, including TKRP, in insects^29–31^. To solve this issue, our further studies concerning the immunomodulatory role of TRP channels will be expanded to include immunocytochemical assays with antibodies targeting markers of cell proliferation and analyses of the relationship between TRP channels and hormone release. Interestingly, dsRNA-based knockdown did not influence the CHC value. These different effects may be the result of the different designs of these two experiments (the CHC value in a group after the *Painless* knockdown was analysed eight days after dsRNA injection). Additionally, the partial discrepancies may be the result of way of Painless channel activation via AITC application. As we mentioned previously, AITC can also activate other TRPA channels in insects, including TRPA1^26^.

The molecular analysis of immune-related genes partially confirmed the role of Painless channels in the regulation of *T. molitor* immune mechanisms. Notably, the responses of the haemocytes and fat body differed, which was highlighted by the significant negative correlation of immune-related gene expression in these two types of cells/tissues. The application of AITC caused the overexpression of immune-related genes in haemocytes, mostly one and three hours after AITC treatment. The observed changes in the expression of *Relish* and *Defensin 1* may indicate a link between TRPA channels and Imd pathways, which is activated during the response to gram-negative bacteria, such as *E. coli*^32^. On the other hand, changes in the expression of *Domeless* may suggest that the modulation of TRPA channels, including Painless, may influence the JAK/STAT pathway. This pathway regulates immune responses and metabolism during infection. The results of the knockdown analysis partially confirmed the direct effect of AITC modulation on the haemocyte Imd pathway in *T. molitor*. After the application of dsRNA, the expression of the *Defensin 1* gene significantly decreased. The connection between calcium signalling and the Imd pathway has not been well explored. However, research by Wei et al.^20^ and Chen et al.^33^ suggested a link between, activated by calcium ions, Calcineurin and the Imd pathway. Our research partially confirms this supposition because the *Calcineurin* gene was significantly overexpressed in *T. molitor* haemocytes three hours after 10^− 2^ M AITC treatment, which is associated with the upregulation of *Relish* and *Domeless* in these cells. Interestingly, research conducted on vertebrates links Calcineurin activity with the JAK/STAT and Toll pathways, which is also partially supported by our research^21,34,35^. However, further research is needed to fully understand this mechanism. For this purpose, our next study will focus on the effect of *Calcineurin* gene knockdown on different TRPA channel functions.

In fat body samples, most of the observed changes were related to the significant downregulation of immune-related genes. These results may indicate that immune mechanisms are inhibited in these tissues. Because TRPA channels, including Painless channels, are also connected to the response of the insect body to unfavourable environmental conditions, AITC injection may indirectly affect insect metabolism through changes in fat body function^7,8^. While this hypothesis is not fully confirmed for insects, research conducted on vertebrates has shown that TRPA channels can be linked, for example, to free fatty acid metabolism^36^. Moreover, recent research by Ahrentløv et al.^29^ points to a close connection between TRPA1 and AKH, the neuropeptide which modulates insect metabolism during the stress response, including during thermal stress^37^. For this purpose, we cannot exclude the possibility that other TRPA channels, such as Painless, may also participate in the regulation of insect metabolism. On the other hand, ds*Painless* treatment led to the simultaneous downregulation of the *Relish*, *Toll*,* Domeless* and *Calcineurin* genes in the fat body, which additionally supports our hypothesis concerning the link between Painless channel and the regulation of immune pathways. Similar to previous results, the observed changes between AITC activity and dsRNA-based knockdown may be connected to the activity of other TRPA channels. For this reason, further analysis is needed to explain the relationships between different TRP channel activities and *T. molitor* fat body functions.

Differences in *T. molitor* physiology were observed not only at the molecular and cellular levels but also at the organismal level. Single injection of AITC and knockdown of *Painless* gene did not significantly affect the survival of *T. molitor*. Interestingly, the 10^− 4^ M AITC injection before the activation of the *T. molitor* immune system via *E. coli* significantly increased the survival rate of the tested beetles. These results may be associated with the results of the molecular analysis conducted after AITC treatment. The upregulation of *Relish* and *Defensin* genes in haemocytes after AITC injection likely allows a more effective response of *T. molitor* against gram-negative bacteria. The changes in the expression of these genes were less pronounced at concentrations of 10^− 2^ M, which may be explained by the moderate effect observed in this group. However, the *Painless* knockdown did not significantly affect *T. molitor* mortality, which suggested that not only Painless but also other TRPA channels activated by AITC may be important for the regulation of the insect immune response^6^. Therefore, our research will continue, particularly in terms of the relationships between different TRP channels in the coordination of the immune response.

In the discussion concerning the value of the obtained results, the possibility of designing a very specific tool that allows the regulation of the *T. molitor* population or the enhancement of insect mass rearing is very important. Phylogenetic analysis revealed that the sequences of the Painless characteristic of Tenebrionidae beetles were clustered in groups separated from those of the other beetle species. These results suggest that owing to the presence of these differences, the modulation of Painless can be a feasible molecular target important in the control of the *T. molitor* population. The differences between the Painless sequences can allow the design of highly specific Painless modulators, which might be helpful in further research on the physiological action of Painless channel. However, this issue needs further investigation.

In summary, our research provides new findings associated with the possible role of one of the most important TRP channels, Painless, in the modulation of the insect immune response. Obtained results suggest that the expression pattern of *Painless* gene changes dynamically during the immune response, which may participate in the regulation of immune activity via the modulation of immune-related pathways, which is likely linked by Calcineurin.

## Materials and methods

### Rearing of *T. molitor*, injection and sample collection

A 7–9-day-old male *T. molitor* was used as a model organism. The beetle culture was reared at the Department of Animal Physiology and Developmental Biology under stable conditions in a MIR 154-PE incubator (PHCbi, Singapore; 28°C, 50–60% RH). Larvae were kept in plastic containers filled with 1.5 kg of oat flakes (500 individuals per box (30 (L) × 15 (W) × 12 (H) cm). Three times a week, the larval diet was supplemented with apples to provide the source of water and vitamins crucial for larval development. Pupae were divided according to sex and kept under conditions similar to those of larval individuals. After pupation, adult males were kept in sterile plastic boxes with compartments. In each compartment, similar amounts of oat flakes and pieces of apple were provided. The use of only young male individuals aims to eliminate fluctuations in immune system activity associated with hormonal changes during oogenesis and changes associated with immunosenescence.

Before injection or sample collection, beetles were anaesthetized with endogenous CO_2_ by immersion in a beaker of clean water for 7 min. Afterward, the beetles were washed in ethanol and distilled water. The tested compounds were injected with a Hamilton microliter syringe (Hamilton Company, Reno, NV, USA) under the coxa of the third pair of legs.

Haemolymph samples were collected by cutting the tibias of the first pair of legs, which allowed us to obtain haemolymph containing only haemocytes without contamination of other cells. The brain, VNC and fat body samples were collected via dissection with a Zeiss Stemi 504 stereomicroscope (Jena, Germany). Before sample collection, microsurgical equipment and all surfaces were cleaned in detail with PCR Clean solution (Minerva Biolabs GmbH, Berlin, Germany). For molecular analysis, samples were collected in RNA lysis buffer (Zymo Research, Irvine, CA, USA). For the physiological analysis, samples were collected in physiological saline (PS, 128 mM NaCl, 18 mM CaCl_2_, 1.3 mM KCl, 2.3 mM NaHCO_3_) or a solution of PS and anticoagulation buffer (4.5 mM citric acid and 9 mM sodium citrate; PS + ACB, 5:1, v/v).

### Activation of the *T. molitor* immune system

To activate the immune system, the procedure previously described by Konopińska et al.^31^ was used. To activate different signalling pathways involved in immune system activation, insects were injected with 2 µL of a suspension of lyophilized *E. coli* K12 (*Ec*, EC1; 1 mg/1 mL in PS; 1.27 × 10^6^ cells/µL; Sigma Aldrich, Saint Louis, MO, USA) or peptidoglycan from *S. aureus* (PG; 77140; 1 mg/1 mL in PS; Sigma Aldrich, Saint Louis, MO, USA) in PS. Moreover, to evaluate the effects of cytokines, the *Tm*Spz-like protein was also used. The protein was synthesized by Biomatik (Kitchener, Canada) on the basis of available sequences and transcriptomic data (https://www.ncbi.nlm.nih.gov). *Tm*Spz-like is among the most important *T. molitor* cytokines closely associated with the Toll pathway^38^. The *Tm*Spz-like solution (2 µL) in PS was injected at a concentration of 10⁻⁷ M. The concentration used was chosen on the basis of the molecular analysis associated with the binding of Spz to the Toll receptor and the structure of the Toll-Spz complex in insects, including the EC_50_ and K_d_ (dissociation constant) values^39,40^. To better visualize the dynamic changes observed in the tested cells, samples were collected at three different time points, 1, 3 and 24 h after the injection of immune system activators. The first two time points may reflect the initial stage of the immune response, whereas the 24-hours research variant is associated with the later phase^41–43^. As a control, *T. molitor* beetles injected with 2 µL of PS were used.

### Expression levels of *Painless* gene

To analyse the expression levels of genes encoding Painless after activation of the immune system (1) and immune-related genes after AITC (2) and dsRNA treatment (3), reverse transcription quantitative PCR (RT–qPCR) was used. In the first case, *T. molitor* brain, VNC, fat body, and haemocyte samples were collected 1, 3, or 24 h after activation of the immune system in the tested individuals. RNA was isolated with a Quick-RNA Mini-prep kit (Zymo Research, Irvine, CA, USA) according to the manufacturer’s protocol, and potential DNA contamination was subsequently removed via a Turbo DNA-free kit (Thermo Fisher Scientific, Waltham, MA, USA). The quality and quantity of the obtained RNA were analysed with a DS-11 spectrophotometer (DeNovix, Inc., Wilmington, DE, USA). Next, equal concentrations of RNA samples (100 ng for the brain, VNC, fat body and haemocytes) were transcribed to cDNA with a LunaScript^®^ RT SuperMix Kit (New England Biolabs, Ipswich, MA, USA). Additionally, a no-RT control was performed to exclude any external contamination of the samples. The sequences of the primers used for the RT‒qPCR assay are summarized in Table 1 (Supplementary materials). Moreover, to confirm the reliability of the primers used, the amplicons were sequenced by the Molecular Biology Techniques Laboratory (Faculty of Biology, Adam Mickiewicz University, Poznań) and compared with data available in the NCBI repository (https://www.ncbi.nlm.nih.gov). RT‒qPCR experiments were performed with a CFX96 thermocycler (Bio-Rad, Hercules, CA, USA). The relative expression of selected genes was normalized to the expression level of the gene encoding *T. molitor* ribosomal protein L13a (*Tm*RpL13a)^22,31,44,45^. Three biological replicates were used for each treatment, and at least two technical replicates were performed. For the brain, VNC, fat body, and tissues pooled from 10 individuals were considered one biological repetition. Owing to the specificity of haemocytes, in one biological repetition haemolymph from 20 individuals was collected (total number of biological samples: 36 variants × 3 biological samples = 108). The relative expression was calculated via the method described by Pfaffl^46^ on the basis of primer efficiency.

**Table 1:**
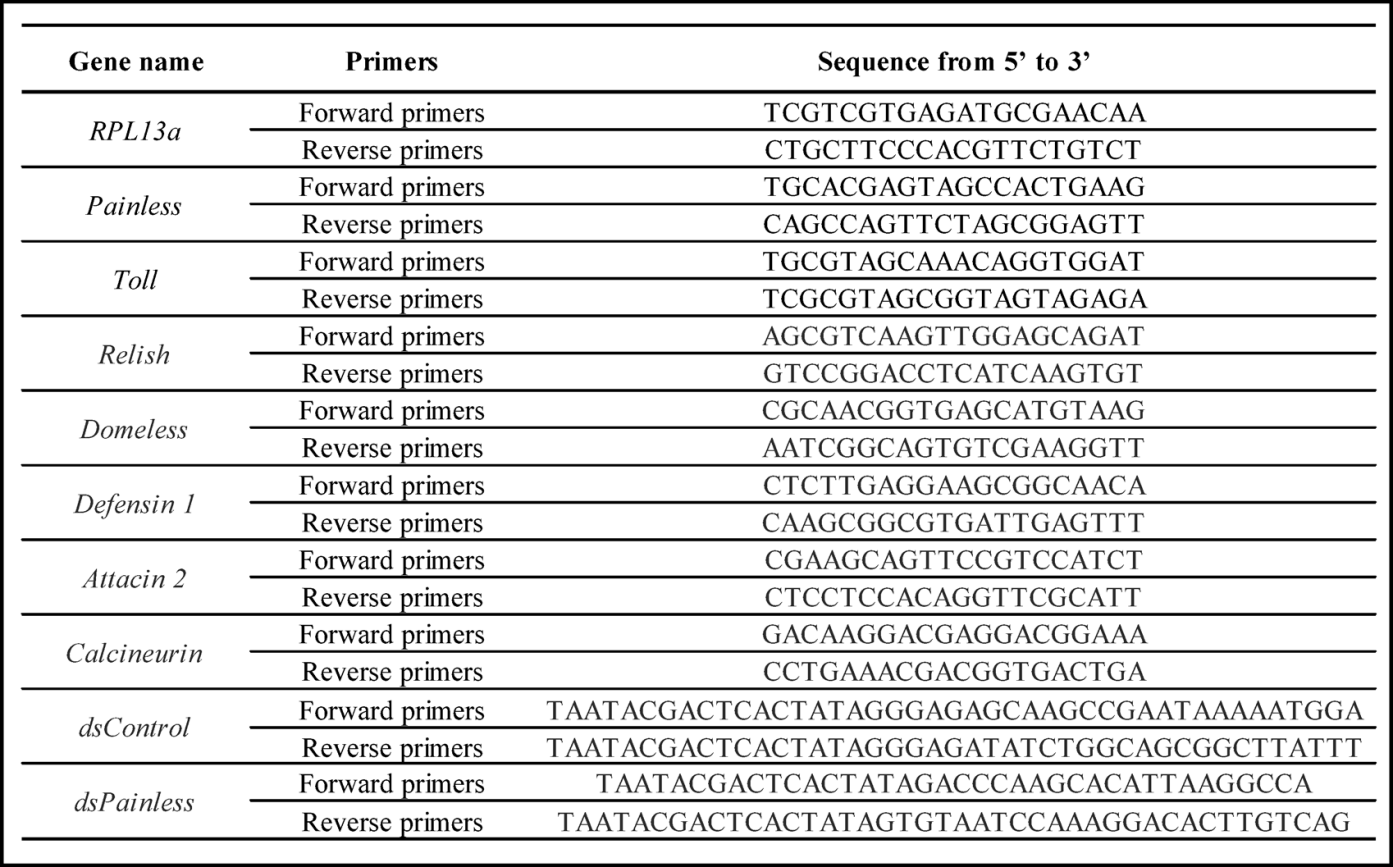
Primers used in the research.

### Potential activation of Painless channels

For the activation of Painless channels, allyl isothiocyanate (AITC, CH2=CHCH2N=C=S) was used (Sigma‒Aldrich, Saint Louis, MO, USA). AITC is a common activator of TRPA channels^8,26^. AITC was dissolved in a 50% solution of EtOH (molecular grade) with extra pure water to obtain a 1 M solution. On the basis of the stock solution, the working solutions were prepared in PS to obtain two concentrations—10^− 4^ and 10^− 2^ M. The concentrations used were based on literature data^26^. Insects were injected with 2 µL of AITC solution. Owing to the presence of 0.5% percent ethanol in the working solution, control individuals were treated with 0.5% ethanol in PS, which did not affect *T. molitor* survival (Fig. 8A).

### Relative calcium level in haemocytes

The relative calcium level in the haemocytes was analysed according to the modified manufacturer’s protocol and methods published by Roy et al.^18^. Haemocytes were chosen as the primary immune cells in this study because of the need to obtain a clean monolayer of immune-related cells. The heamolymph sample (2 µL) was added to 20 µL of PS and mixed gently by pipetting. The samples were subsequently placed on clean, ethanol-washed, microscopic slides and incubated for 30 min in the dark. After incubation, the residues of the sample were removed, and the microscopic slide was washed three times with PS. Next, the samples were incubated with a solution of 20 µM Fluo-8 AM (Abcam, Cambridge, UK) supplemented with 0.04% Pluronic F-127 (Sigma‒Aldrich, Saint Louis, MO, USA) in PS for one hour in the dark. After incubation, the sample was washed three times with a solution of 1% Probenecid (Sigma‒Aldrich, Saint Louis, MO, USA) in PS. The haemocyte samples were subsequently incubated with a solution of AITC and Probenecid in PS (0.5% EtOH) for 15, 30–45 min. In the experiment, two concentrations of AITC were used—10^− 4^ and 10^− 2^ M. Control individuals were incubated with a solution of Probenecid in PS supplemented with 0.5% EtOH. In the next step, the haemocytes were fixed with a 4% solution of paraformaldehyde in PS for 15 min. Next, the samples were washed three times with PS and mounted with glycerol for fluorescence microscopy. The samples were examined with a Nikon Eclipse TE fluorescence microscope equipped with a Nikon DS-1QM digital camera. From each sample, 5 random micrographs were taken. The fluorescence intensity is an indicator of the calcium level in haemocytes. The performance of the control samples was always the same as that of the experimental samples. Moreover, all micrographs were taken under the same microscopic conditions. The relative calcium level was determined on the basis of the fluorescence intensity via the protocol described by Saito and Mori^47^ with ImageJ software (version 2.0). At least 9 individuals were used in each research variant. The experiments were repeated at least 3 times. (Control 15 min – 14 beetles; AITC 10^− 4^ M 15 min – 11 beetles; AITC 10^− 2^ M 15 min – 11 beetles; Control 30 min – 13 beetles; AITC 10^− 4^ M 30 min – 13 beetles; AITC 10^− 2^ M 30 min – 9 beetles; Control 45 min – 10 beetles; AITC 10^− 4^ M 45 min – 10 beetles; AITC 10^− 2^ M 45 min – 11 beetles).

### Circulating haemocytes count

The circulating haemocyte count (CHC) was analysed via the method previously described by Bruno et al.^48^ and Urbański et al.^28.^ The haemolymph sample (2 µL) was mixed with 20 µL of a solution of PS and anticoagulation buffer. Afterward, 7 µL of sample was placed in FAST READ 102 counting chambers (Biosigma S.R.L., Italy) and analysed via a Zeiss Primostar microscope equipped with an AxioCam 105 digital camera (Zeiss, Jena, Germany). For each sample, photos of 24 randomly selected squares were taken and analysed via ImageJ software (version 2., public domain, https://imagej.net/software/imagej2/). At least 10 individuals were used per treatment (Control 1 h – 14 beetles; AITC 10^− 4^ M 1 h – 14 beetles; AITC 10^− 2^ M 1 h – 13 beetles; Control 3 h – 14 beetles; AITC 10^− 4^ M 3 h – 10 beetles; AITC 10^− 2^ M 3 h – 11 beetles; Control 24 h – 17 beetles; AITC 10^− 4^ M 24 h – 17 beetles; AITC 10^− 2^ M 1 h – 13 beetles).

### Expression levels of immune-related genes

To determine the changes in the expression levels of selected immune genes after AITC injection, molecular analysis was performed on fat body and haemocyte samples on the basis of the protocol described in the section *“Expression levels of Painless gene*. As one repetition, samples collected from 10 (fat body) or 20 individuals (haemocytes) were considered. The sample preparation protocol was similar to the protocol described above (total number of samples after AITC treatment: 18 variants x 3 biological samples = 54). On the basis of the literature and our previous research concerning the hormonal regulation of the *T. molitor* immune response, as an indicator of the activation of basic immune pathways, we determined the changes in the expression levels of the gene encoding Relish, a transcription factor crucial for Imd signalling, and the gene encoding the Toll receptor, which is an important part of the Toll pathway, and Domeless, the receptor being a part of the JAK/STAT pathway. Moreover, the expression levels of genes encoding two AMPs (Attacin 2 and Defensin 1) were also estimated. The sequences of the primers used for the RT‒qPCR assay are presented in Table 1.

### Knockdown of Painless gene

The procedure for the knockdown of the *Painless* gene was based on a modified protocol by Keshavarz et al.,^49^, Konopińska et al.^22^, and Zanchi et al.^50^. Total RNA was extracted from adult *T. molitor* and *G. mellonella* larvae (control) via the Quick-RNA MiniPrep Kit. As an internal control for the influence of dsRNA injection on *T. molitor* beetles, dsRNA targeting the gene encoding *G. mellonella* lysozyme (*GmLys*), which lacks sequence homology to the known *T. molitor* gene, was used. The extracted RNA was analysed with a TurboDNA-free Kit, and the concentration and integrity of the extracted RNA were analysed with a DS-11 spectrophotometer. Next, cDNA was synthesized via the LunaScript^®^ RT SuperMix Kit. To confirm the RNA purity and quality, a no-RT control experiment was performed. The target fragments were amplified from the cDNA via the KAPA2G Fast ReadyMix PCR Kit (KAPA Biosystems, Sigma‒Aldrich, St. Louis, MO, USA) with the primers listed in Table 1. Amplicon quality was verified by electrophoresis on 2% TAE agarose gels stained with ethidium bromide. The PCR products were subsequently purified via a PCR/DNA Clean-Up Kit (EURx, Gdańsk, Poland). dsRNA synthesis was performed with a High-Yield T7 RNA Synthesis Kit (Jena Bioscience, Jena, Germany) following the manufacturer’s protocol, with incubation at 37 °C for 4 h. Potential residual DNA was removed by additional TurboDNAase treatment after the dsRNA synthesis step. For RNA hybridization, the samples were heated to 95 °C and cooled overnight in a thermocycler. The obtained dsRNA was purified via sequential washes with 5 M NH₄OAc (Thermo Fisher Scientific, Waltham, MA, USA) and an ethanol gradient (70–99%, molecular grade; Bioultra). The final dsRNA pellet was resuspended in nuclease-free water and stored at − 20 °C until use.

**Fig. 10:**
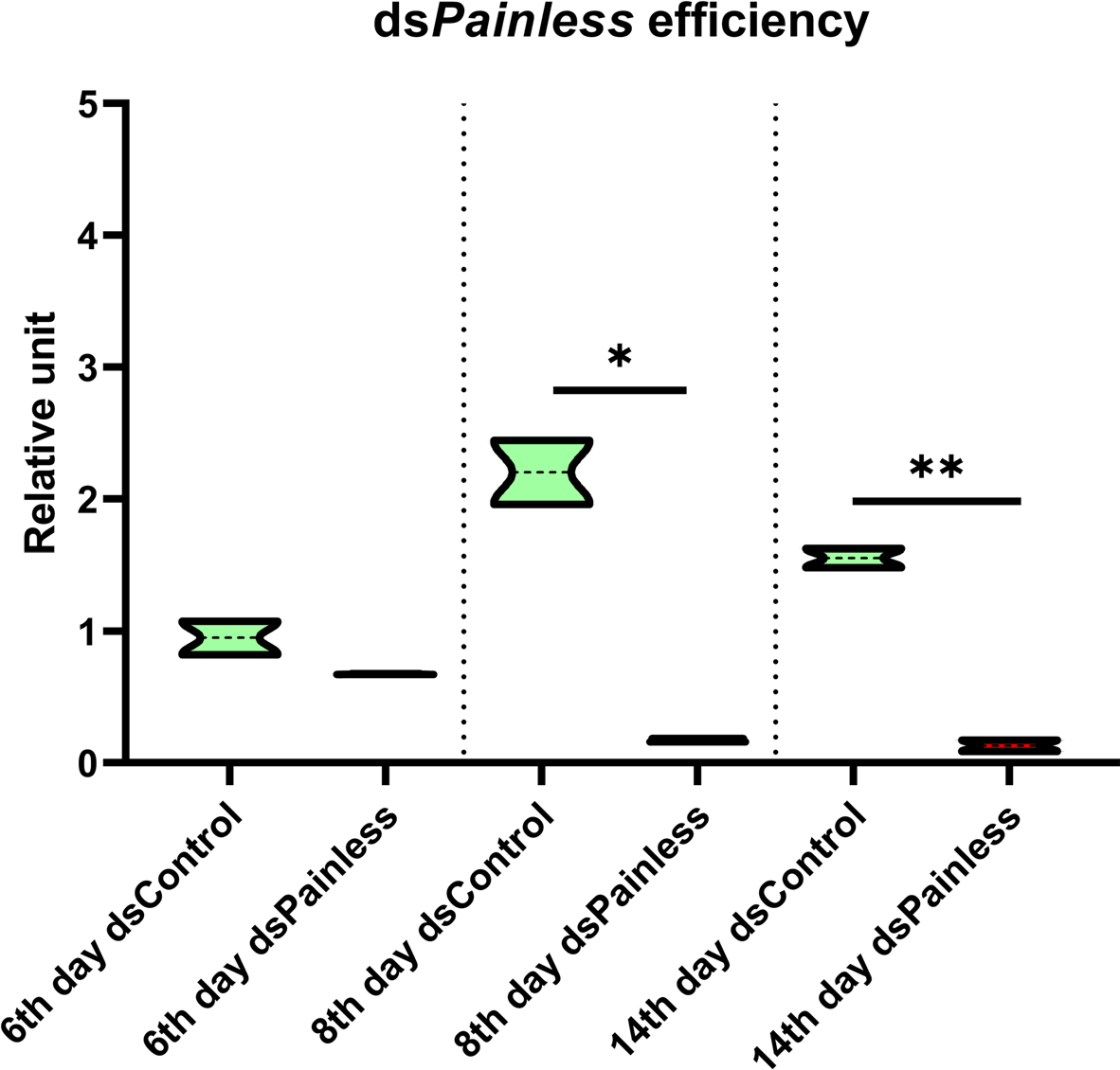
Violin plot of the knockdown efficiency at day 6^th^, 8^th^ and 14^th^. dsControl - control individuals for the dsRNA experiment; beetles were injected with the dsRNA-targeted gene encoding *Galleria mellonella* lysozyme (*GmLys*). ds*Painless* – beetles after the knockdown of *Painless* gene; * *p* ≤ 0.05, ** *p* ≤ 0.01.

*Painless* gene knockdown was achieved via the injection of beetles with 2 µg of obtained dsRNA (1 µg/µL in 2 µL of water). The knockdown efficiency was evaluated prior to the experiments by extracting total RNA (*n* = 3 pools of three adults per day) at multiple time points after injection with dsRNA (Fig. 9), followed by RT–qPCR as described above. The time point (8^th^ day) corresponding to stable transcript silencing was subsequently selected for experiments (Fig. 10).

On the 8^th^ day after the injection of dsRNA, fat body and haemocyte samples were collected as described in the section *‘Expression levels of Painless gene’*. As one repetition, samples collected from 10 (fat body) or 20 individuals (haemocytes) were considered (total number of samples: 4 research variants × 3 biological samples = 12).

### Survival experiment

The survival experiments were performed following the protocol described by Konopińska et al.^22^. After the injection of 0.5% ethanol in PS (control), AITC at concentrations of 10^− 4^ or 10^− 2^ M (0.5% ethanol in PS), dsControl or ds*Painless*, 10 males were kept in a plastic box for 21 days. In the case of the dsRNA experiment, owing to the results of the knockdown efficiency test, survival analysis started 8 days after dsRNA injection. The beetles were kept under the same conditions as the other individuals used in this study. The number of dead and living individuals was checked three times a week during the experiment. Dead individuals were removed from the boxes. Ten individuals kept in plastic boxes were considered one biological replicate. The additional variant was used for survival experiments with simultaneous activation of the *T. molitor* immune system. Two hours after the injection of 2 µL of PS or AITC at concentrations of 10^− 4^ or 10^− 2^ M, the beetles were injected with 2 µL of *E. coli* K12 (1 mg/1 mL in PS; 1.27 x 106 cells/µL). In the case of *Painless* knockdown, the tested individuals were injected with an *E. coli* suspension 8 days after dsRNA application. Each research variant was repeated at least five times (5 × 10 individuals = 50 beetles per treatment).

### Phylogenetic analysis

Phylogenetic analysis of Painless channel was conducted based on the data retrieved from the NCBI database (https://www.ncbi.nlm.nih.gov). A phylogenetic tree was created with Tree Viewer (https://www.ncbi.nlm.nih.gov/tools/treeviewer/). The phylogenetic distance was calculated based on the Grishin method (fast minimum evolution, max sequence difference = 0.85). For phylogenetic tree visualization, iTOL software was used (https://itol.embl.de).

### Statistical analysis

Statistical analysis was performed via GraphPad Prism 9 software (Adam Mickiewicz University licence). The outliers were identified via the ROUT method. The normality of the distribution was tested via the Shapiro‒Wilk test. Depending on the number of tested research variables, results consistent with the normality of the distribution were analysed by one-way ANOVA with Dunnett’s *post hoc* test or Student’s *t*-test (depending on the number of analysed groups). The results with a nonnormal distribution were analysed with the Kruskal‒Wallis test with Dunn’s *post hoc* test and the Mann‒Whitney U test (depending on the number of analysed groups). The correlation of the data was analysed via the Pearson correlation coefficient method in the SRplot software (https://www.bioinformatics.com.cn/srplot). In the manuscript, only statistically significant correlations were described. Survival curves were generated on the basis of the Kaplan‒Meier estimator. The differences between survival curves were calculated via the Gehan–Breslow–Wilcoxon test.

## Supplementary Information

Below is the link to the electronic supplementary material.

Supplementary Material 1

## Data Availability

The datasets used during the current study are available from the corresponding author upon request.
