# Supplementary material for: A possible role of Painless channel in the regulation of immune system activity in Tenebrio molitor L
Source: Sci Rep. 2026 Apr 2;16:15454. doi: 10.1038/s41598-026-45339-x (PMC13184108; doi:10.1038/s41598-026-45339-x)
Supplement: Supplementary file 1 — Supplementary Material 1 [file 41598_2026_45339_MOESM1_ESM.docx]

**Supplementary materials: A possible role of Painless channel in the regulation of immune system activity in *Tenebrio molitor* L.**

Natalia Bylewska^#1^, Radosław Gmyrek^#1^, Natalia Konopińska^1^, Sara Tchórzewska^1^, Grzegorz Nowicki^2^, and Arkadiusz Urbański^1^*

^#^ -equal contribution, shared first authorship

^1^ Department of Animal Physiology and Developmental Biology, Faculty of Biology, Adam Mickiewicz University, Poznań, Poland

^2^ genXone S.A. Złotniki, Poland

* corresponding author: Arkadiusz Urbański, email: arur@amu.edu.pl





**Fig. S1**. The changes in the expression level of *Painless* gene in brain, ventral nerve cord (VNC), fat body and haemocytes 1, 3, and 24 hours after activation of immune system of *Tenebrio molitor* males. Control – individuals injected with physiological saline; Ec – beetles injected with suspension of *Escherichia coli* (OD_600_=1) in physiological saline. PG – beetles injected with suspension of peptidoglycan of *Staphylococcus aureus* (OD_600_=1); Spatzle – individual injected with solution of physiological saline and Spätzle-like protein at concentration of 10^-7^ M. The violin plot presents the data distribution; # 0.05 ≤ *p* ≤0.1; * *p*≤0.05, ***p*≤0.01, ****p*≤0.001, *****p*≤0.0001

**

****Fig. S2.** The changes in expression level of immune-related genes in haemocytes of *T. molitor* after injection of allyl isothiocyanate (AITC) at concentrations of 10^-4^ and 10^-2^ M. Control – individuals after injection of physiological saline. Samples were collected 1, 3 and 24 hours after the injection of AITC. The violin plot presents the data distribution; # 0.05 ≤ p ≤0.1;, ***p*≤0.01, ****p*≤0.001.



**Fig. S3**. The changes in expression level of immune-related genes in fat body of *T. molitor* after injection of allyl isothiocyanate (AITC) at concentrations of 10^-4^ and 10^-2^ M. Control – individuals after injection of physiological saline. Samples were collected 1, 3 and 24 hours after the injection of AITC. The violin plot presents the data distribution. # 0.05 ≤ *p* ≤0.1, **p*≤0.05, ***p*≤0.01.



**Fig. S4**. The changes in expression level of immune-related genes in haemocytes and fat body of *T. molitor* after dsRNA-based knockdown of *Painless* gene. dsControl – individuals after injection of physiological saline. dsControl – beetles injected with a dsRNA-targeted gene encoding *G. mellonella* lysozyme (*GmLys*). The violin plot presents the data distribution. # 0.05 ≤ *p* ≤0.1, **p*≤0.05, ***p*≤0.01.





**Fig. S5.** The violin plots represent the changes in the expression level of *Calcineurin* gene in haemocytes and fat body of *T. molitor* after injection of allyl isothiocyanate (AITC) at concentrations of 10^-4^ and 10^-2^ M, and dsRNA-based knockdown of *Painless* gene. Control – individuals after injection of physiological saline. Samples were collected 1, 3 and 24 hours after the injection of AITC or physiological saline. dsControl – beetles injected with a dsRNA-targeted gene encoding *G. mellonella* lysozyme (*GmLys*). ds*Painless* – beetles injected with dsRNA targeted the *Painless* gene * *p*≤0.05, ***p*≤0.01
